# Revolutionizing the Use of Honeybee Products in Healthcare: A Focused Review on Using Bee Pollen as a Potential Adjunct Material for Biomaterial Functionalization

**DOI:** 10.3390/jfb14070352

**Published:** 2023-07-04

**Authors:** Arka Sanyal, Anushikha Ghosh, Chandrashish Roy, Ishanee Mazumder, Pasquale Marrazzo

**Affiliations:** 1 School of Biotechnology, KIIT Deemed University, Bhubaneswar 751024, India; 1760038@kiitbiotech.ac.in (A.S.); 1860034@kiitbiotech.ac.in (A.G.); 1860063@kiitbiotech.ac.in (C.R.); 1860080@kiitbiotech.ac.in (I.M.); 2 Department of Medical and Surgical Sciences, University of Bologna, 40126 Bologna, Italy

**Keywords:** honeybee products, bee pollen, biomaterials, functionalization, biomedical materials, functionalized materials, regenerative medicine, tissue engineering

## Abstract

The field of biomedical engineering highly demands technological improvements to allow the successful engraftment of biomaterials requested for healing damaged host tissues, tissue regeneration, and drug delivery. Polymeric materials, particularly natural polymers, are one of the primary suitable materials employed and functionalized to enhance their biocompatibility and thus confer advantageous features after graft implantation. Incorporating bioactive substances from nature is a good technique for expanding or increasing the functionality of biomaterial scaffolds, which may additionally encourage tissue healing. Our ecosystem provides natural resources, like honeybee products, comprising a rich blend of phytochemicals with interesting bioactive properties, which, when functionally coupled with biomedical biomaterials, result in the biomaterial exhibiting anti-inflammatory, antimicrobial, and antioxidant effects. Bee pollen is a sustainable product recently discovered as a new functionalizing agent for biomaterials. This review aims to articulate the general idea of using honeybee products for biomaterial engineering, mainly focusing on describing recent literature on experimental studies on biomaterials functionalized with bee pollen. We have also described the underlying mechanism of the bioactive attributes of bee pollen and shared our perspective on how future biomedical research will benefit from the fabrication of such functionalized biomaterials.

## 1. Introduction

**Figure 1:**
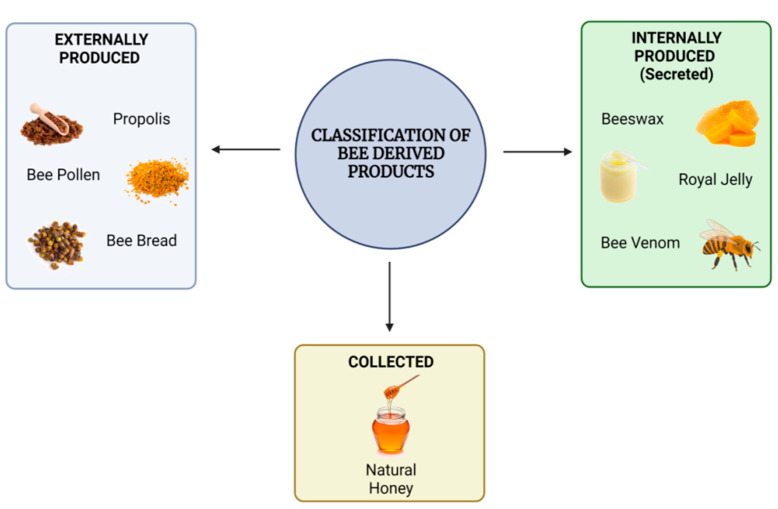
Classification of honeybee-derived products.

Humanity is entirely reliant on natural resources for survival. Nature provides the energy that sustains our cells, the nutrients that build up human bodies, and the ecosystem comprising biodiversity from which our day-to-day necessities are derived. According to specialists at the United Nations Food and Agriculture Organization (FAO), bees are responsible for one-third of the world’s food output. Bees are ideally designed to pollinate, aiding plants in growing, reproducing, and producing food by transporting pollen between flowering plants and keeping the life cycle going. Additionally, they produce high-quality food, such as honey, but also functional food and superfoods, such as royal jelly, propolis, bee pollen, bee bread, and other materials, such as beeswax and bee venom, all of which have different nutraceutical and medical applications within our current lifestyle. Honeybee products can typically be classified into two distinct types based on how they are collected: produced externally or produced internally and then secreted, as shown in Figure 1.

### 1.1. Honeybee-Derived Products

Among honeybee-derived products, natural honey remains the most common. It is typically a sweet, viscous, golden-colored liquid produced by honeybees as a by-product of floral nectar, which the bees store in their hives for subsequent use as a source of nutrition. Its complex chemical composition, color variety, and flavor change depending on the source of the honey’s botanical ingredients [1]. Although it is most frequently used as a sweetener, it has traditionally been recognized for some therapeutic properties. It is a blend of phytochemicals with a history of having effective antibacterial, anti-inflammatory, and antioxidant agents [2]. Worker bees initially collect nectar from a flower using their proboscis and store it in their upper aerodigestive tract, known as the bee crop, to transport the nectar to the hive. Within the bee crop, the nectar, which contains complex carbohydrates, interacts with enzymes generated by specific glands, resulting in its degradation [3]. Once the worker bees reach the hive, another group of bees, known as house bees, collect the nectar from the worker bees and start processing it using the enzymes invertase and glucose oxidase, resulting in a change in the composition and pH of the nectar [4]. Gradually, the sugar molecules begin to transform, resulting in the formation of gluconic acid and hydrogen peroxide [3]. These molecules are primarily responsible for the acidic and antibacterial properties of natural honey. The surplus water in the plant nectar is then eliminated by exposing it to the warm, dry air within the hive. To boost airflow through the hive, bees spread their wings. This continuous fanning aids in reducing the moisture content of the nectar through evaporation. This dehydration process concentrates the honey, resulting in a supersaturated sugar solution containing organic acids, minerals, amino acids, vitamins, phenols, and other minor substances [5].

The most essential component of the diet of honeybee larvae is royal jelly, a secretion product of the nurse bees’ cephalic glands and crucial for caste differentiation [6]. Worker bees secrete royal jelly, a white, viscous jelly-like fluid, from their mandibular and hypopharyngeal glands. It comprises water (50–60% *w*/*w*), proteins (18% *w*/*w*), carbohydrates (15% *w*/*w*), lipids (3–6% *w*/*w*), mineral salts (1.5%), and vitamins, hence being referred to as a “superfood” that is consumed by the queen bee throughout her life, allowing her to grow larger than the other bees. When honeybee larvae hatch, they are given royal jelly, which helps to nourish the brood during their initial few days of maturity [7]. Besides this, the Major Royal Jelly Proteins (MRJPs) are known to play an essential nutritional role in the queen bee’s diet [8].This superfood is the major reason why the queen bee lives longer than the other bees. Furthermore, royal jelly has been found to contain a family of peptides known as jelleines, which are reported to possess antimicrobial properties in vitro [9].

Propolis, often known as bee glue, is a resinous substance produced by worker bees by combining saliva and beeswax with exudate collected from tree buds, sap flows, or other botanical sources [10]. It is mainly associated with protective functions for the beehive. Literally, it means “hive defense material,” which can be translated from the Greek words “pro”, meaning “in front of”, and “polis”, which means “city” [11]. It is utilized to seal up any undesired open spaces or fractures present in the beehive. In addition, propolis is used to smooth the interior surface, maintain thermal insulation to regulate the optimum internal temperature of the beehive, reduce water loss from the hive, prevent weathering (deterioration) of the hive, and protect against predator invasion [12]. It also contributes to maintaining an aseptic interior environment due to its antibacterial properties and is used to cover the carcasses of deceased bugs and other predators that have penetrated the hives.

Bee pollen, commonly known as ambrosia, is typically a pellet of field-collected flower pollen packed by worker honeybees and utilized as the hive’s principal food supply. Worker bees gather pollen from anthers present in flowers, mix it with a small number of salivary gland secretions or nectar, and deposit it in hairy receptacles (corbiculae), commonly known as pollen loads, present on the tibia of their hind legs [13]. Bee pollen is collected and transported to the hive by worker bees, where it is deposited in brood cells, combined with saliva, and sealed with a drop of honey [14].

Beeswax is a complex substance secreted in liquid form by particular wax-producing mirror glands on the inside surfaces of the sternites on the abdominal segments of younger worker bees aged between 12 and 18 days [15]. The activity of the wax glands declines in aged bees but can be restarted in an emergency. Most beeswax is produced during the bee colony’s growth period in temperate climate conditions [16]. Once it comes into contact with air, it solidifies into a waxy material, which the bees reshape with their jaws to build the honeycombs by adding pollen and propolis. The hive worker bees gather it and utilize it to construct chambers within the beehive for storing honey as well as for the protection of larvae and pupae. Pure beeswax is practically colorless when secreted by the bee. However, over time, after contact with honey and pollen, it assumes a strong yellowish hue, eventually becoming brown since it includes the cocoon [17].

Bee bread is a natural substance and the primary food source for honeybees. It is made by fermenting bee pollen, bee saliva, and floral nectar inside the honeycomb cells of a hive [18]. During storage within the honeycomb, bee pollen is subjected to digestive enzymes and undergoes lactic acid fermentation to create bee bread. The specific content of the bee bread changes not only based on the plants foraged by the bees but also across regions, seasons, and even at various times of day [19].

Bee venom, also known as apitoxin, is a colorless, acidic liquid that is secreted by bees through their stingers into a target in self-defense. It is produced in poison glands in the abdominal cavity of female worker bees (male bees do not possess these glands) [20].

All these products have reportedly been found to be storehouses for various bioactive compounds. The medicinal and healthcare benefits associated with bee-derived products are an attribute of these bioactive molecules. While a promising number of such molecules have successfully been demonstrated to have health benefits, others are still being researched. There are different bioactive compounds found in various honeybee-derived products. A summary of these bioactive compounds reportedly found in each of the known bee-derived products has been summarized in the table given below, Table 1.

### 1.2. Medicinal Value of Honeybee Products for Human Health

The exploitation of honeybee products by humans for medicine and therapy has been going on since time immemorial. Honey and other bee products have been used in human therapies for thousands of years, and their healing abilities are even mentioned in various religious texts [29]. Beekeeping, also referred to as apiculture, is the husbandry of bee colonies where beekeepers raise bees in man-made beehives to harvest hive contents. These items, or apiproducts, besides having a high nutritional value, possess significant potential, as a result of which they have frequently been employed in several countries as complementary medicinal agents. This type of integrative therapy involving the utilization of bee-derived products to treat various health conditions has been referred to as “apitherapy” [30]. In essence, apitherapy is a type of complementary medicine in which bee-derived products such as honey, pollen, propolis, royal jelly, and bee venom are used to treat diseases and their symptoms and pain from acute and chronic injuries. There are different extensions of apitherapy depending on the particular bee product used and the clinical condition in question. The clinical applications of these products are widespread. However, the suitability of natural honey for wound healing applications remains the most well-studied application for an apiproduct to date.

Indeed, the therapeutic efficacy of natural honey has been described in the world’s earliest medical literature, and it has been demonstrated to be effective on multiple wound types [31]. The wound-healing properties of natural honey stem from its antibacterial activity, its ability to keep wounds moist, and its high viscosity, which helps establish a barrier against infection. The antibacterial properties of honey are attributed to its high sugar concentration that dehydrates bacteria by osmosis; its acidic pH ranging between 3.2 and 4.5, which is low enough to prevent the growth of most microbes; the production of hydrogen peroxide, which has a germicidal effect, from glucose present in the enzyme glucose oxidase; and other phytochemical and bioactive components [discussed in the previous section] that impart honey’s immunomodulatory properties. Owing to its high sugar content, natural honey can help reduce the amount of water on the wound bed, creating a moist environment conducive to healing [24]. Besides, honey has been shown to stimulate collagen production and promote fibroblast infiltration, both of which are particularly important during the proliferative phase of wound healing [32,33]. While many different forms of natural honey exist, one particular form needs special mention due to its superior performance as a wound healing material. Manuka honey (MH), in particular, has been reported to have very significant antibacterial action, which is attributed to the presence of methylglyoxal (MGO), a glycation inducer, and is generated by bees that pollinate the Manuka tree (*Leptospermum scoparium*) in New Zealand [24]. MGO is produced by the non-enzymatic conversion of dihydroxyacetone, which is abundant in the nectar of the *L*. *scoparium* flower. When used as a wound dressing, MH helps keep the wound moist and functions as an autolytic debriding agent for wounds [34]. In their study, Gethin et al. reported that MH was found to be helpful in reducing wound size and promoting wound healing in patients with leg ulcers [35]. Overall, the data demonstrate that MH has the ability to improve wound healing in people, especially those with leg ulcers. However, more study is needed to fully comprehend the processes by which it stimulates wound healing and evaluate its efficacy in various types of wounds. Several manufacturers are now providing or developing honey products (mostly utilizing MH) for the treatment of wounds, ulcers, and burns. Derma Sciences, a firm specializing in the production of Food and Drug Administration (FDA)-approved formulations for wound care, manufactures a range of MH products under the trademark Medihoney^TM^ [32].

Propolis is another beehive-derived product that has been investigated for its possible role in the wound healing process and has shown promising outcomes as a therapeutic agent for other clinical conditions. The bioactive phytochemical constituents of propolis provide immune defense besides having antimicrobial and antioxidant properties. Given that propolis’ function is to support beehive sterility and health, the protective properties of the bioactive compounds found in propolis can provide significant benefits for human health [36]. Consequently, propolis has numerous biological characteristics, making it an appropriate component for several therapeutic interventions. Propolis has been shown in numerous studies to have therapeutic potential in pharmacies and medicines to treat a variety of chronic diseases, especially autoimmune diseases, diabetes, burns, wounds, gynecological issues, dermatological, neurodegenerative, gastrointestinal, cardiovascular, and respiratory tract-related diseases [36]. Even though the mechanism by which propolis promotes health appears to be related to its antioxidant and inflammatory activity, the range and scope of physiological effects of this multifaceted nutrient are extensive and varied in most cases. Propolis has antibacterial, antiviral, antifungal, and anti-inflammatory properties. This promotes its use in dentistry for various procedures such as root canal maintenance, tooth storage for avulsed teeth, direct and indirect pulp capping, caries prevention, and accelerating the recovery from surgery wounds [37]. According to reports, propolis can overcome the bacterial resilience developed by types of bacteria like *Enterococcus faecalis* and *Staphylococcus aureus*. Additionally, it has been noted that when propolis comes into contact with exposed pulpal tissues, ethanolic propolis extract induces the creation of dentin bridges and bone regrowth [38]. The propolis constituent apigenin and many other flavonoids have been reported to have anti-diabetic properties [39]. Naringin, another naturally occurring flavanone glycoside present in propolis, has also been noted to have characteristics similar to insulin and lipid-reducers that lower glucose and insulin resistance [39]. Apigenin and naringin have both been used to boost the suppression of the glycogen phosphorylase enzyme and glucose absorption by muscle cells, thereby lowering blood sugar levels [36]. According to several studies, propolis’ molecular constituents, which include terpenoids, phenolics, steroids, alcohols, terpenes, and sugars, are capable of curing rheumatoid arthritis. Brazilian propolis has been shown to be effective in lowering the activity of RA disease in mice, indicating that propolis may offer new therapeutic alternatives [40]. Additionally, propolis has also been linked to a role in the healing of motor neurons and myelinated fibers, alleviating symptoms of brain and neurological disorders and injuries [41]. Moreover, propolis can reduce reactive oxygen species (ROS) by increasing antioxidants, and NF-κB pathways, and suppressing the inflammatory cascade [42]. Interestingly, flavonoids, which are among the active components present in propolis, have chemopreventive properties against the majority of cancer types [43]. According to recent clinical research, propolis may help combat COVID-19 by efficiently blocking the human body’s SARS-CoV-2 helicase in patients and helping them feel better and receive more effective therapy [44].

One of the honeybee products with promise for treating a variety of human diseases is royal jelly because of its bioactive constituents. Proteins, peptides, lipids, phenolics, and flavonoids have been identified as the primary bioactive compounds responsible for the various health benefits of royal jelly [45]. Due to the presence of specific proteins and antimicrobial peptides (e.g., MRJPs), royal jelly has strong antimicrobial properties against various pathogens. Furthermore, royal jelly has the ability to combat periodontopathic bacterial species such as *Aggregatibacter actinomycetemcomitans*, *Fusobacterium mucleatum*, *Prevotella intermedia*, and *Porphyromonas gingivalis* [46]. Furthermore, native jelleines found in royal jelly have been shown to inhibit the growth of both gram-positive bacteria (*Bacillus subtilis* and *Staphylococcus aureus*) and gram-negative bacteria (*Escherichia coli* and *Pseudomonas aeruginosa*) known to cause wound infections [45]. This demonstrates their utility as antimicrobial agents in promoting wound healing by preventing infection. Under the influence of royal jelly, human fibroblasts were able to spread and raise levels of sphingolipids by reducing the secretion and production of collagen in both in vivo and in vitro wound-healing models. As a result, royal jelly accelerated the healing of desquamated skin sores [47]. In addition to conventional therapies, royal jelly dressing is a successful method of managing diabetic foot ulcers. This is attributed to its vasodilation effects, which help to improve blood flow around the impacted area [48]. Royal jelly has excellent antioxidant properties that have been investigated for both the prevention and therapy of a variety of chronic and degenerative diseases. In one such study, evidence regarding the neuroprotective properties of royal jelly was reported, where the administration of royal jelly reduced oxidative stress, neuronal death, and biological markers of Alzheimer’s disease while improving cognitive impairments in rodent models [49]. Interestingly, 10-hydroxy-trans-2-decenoic acid (10-HDA), a bioactive component found in royal jelly, has been found to stimulate the fibroblast’s production of collagen. By boosting the skin’s collagen production through epidermal growth factor (EGF) signaling, royal jelly exhibits anti-aging effects by preventing fine lines, wrinkles, and skin sagging [45]. Recently, royal jelly has been realized to improve cardiac function through its anti-hypercholesterolemic effects, and its administration has been correlated with decreased levels of cholesterol and lipids in serum [50]. Moreover, royal jelly has been shown in randomized clinical research in postmenopausal women to successfully treat urinary issues and improve life quality [51]. Other notable health benefits reported using royal jelly include decreased blood glucose levels in diabetics, where its administration into the physiological system results in increased insulin production [52], and cancer prevention via inhibition of tumor growth and/or metastasis, by the inhibition of tumor-induced angiogenesis, and/or the activation of immune function [53].

Bee pollen is utilized in apitherapeutic applications owing to its anti-fungal, antibacterial, antiviral, anti-inflammatory, immunostimulating, antioxidant, and local pain relief properties, besides being known to facilitate the granulation phase of burn wound healing [25]. Research has linked the phenolic acid and flavonoid content of bee pollen to its antimicrobial activity. The breakdown of the cytoplasmic membrane, which results in the release of potassium ions and the onset of cell autolysis, is the mechanism by which flavonoids and phenols exert antibacterial, and anti-fungal behavior [54]. The flavonoids, steroids, and volatile oil molecules in bee pollen are thought to be responsible for their immunosuppressive effects. According to research by Ishikawa et al., bee pollen may influence both the early and late phases of allergic responses by decreasing mast cell activation and enhancing the anti-allergic response [55]. The principal inhibitors of the allergenic response, histamine production, and suppression of IgE binding to its receptor (FcRI) are how the anti-allergic properties of bee pollen are demonstrated [56]. The ability of bee pollen bio-compounds to activate or inhibit protein phosphorylation and consequently affect cell signaling pathways, including the regulation of cell proliferation, may be another significant mode of action on cell function. Fatty acids and phytosterols, which are active in the anti-inflammatory process, are also linked to the anti-inflammatory mechanism of bee pollen [57]. Bee pollen’s antioxidant properties may be due to the action of antioxidant enzymes as well as the presence of secondary plant metabolites such as phenolic compounds, carotenoids, vitamin C, vitamin E, and glutathione [58]. It has been demonstrated that the flavonoids found in bee pollen may neutralize electrophiles, scavenge free radicals, and ROS, and as a result, stop them from becoming mutagens [54]. Additionally, blood sugar levels are improved by bee pollen, with studies reporting that aqueous-ethanol extracts and water extracts of bee pollen can naturally act as regulators of α-amylase and β-glucosidase, respectively, to lower blood sugar levels [59]. Bee pollen has also been speculated to have a preventive effect against diabetes-induced pituitary testicular dysfunctions and the associated detrimental changes due to its antioxidant defense systems [60]. The high concentration of phenolic compounds in bee pollen may play a role in avoiding obesity and associated health concerns. Additionally, in vivo experimental findings from rats and rabbits have revealed that pollen has hypolipidemic action, lowering plasma total lipids and triacylglycerols. Moreover, pollen consumers have a decreased ability to aggregate platelets and an enhanced fibrinolytic system activity, implying the anti-atherosclerotic property of bee pollen that protects against heart disease and stroke [61]. According to recent studies, bee pollen possesses anti-allergic properties. It shields the body’s mast cells from degranulation, which results in the release of histamine, which is the mediator of allergic reactions.

Bee pollen is also known to reduce uric acid levels. Studies report that rapeseed bee pollen has the ability to be used clinically as an anti-hyperuricemia agent [62]. The hepatoprotective role of bee pollen has also been reported, whereby by boosting the activities of glutathione-S-transferase (GST), glutathione (GSH), superoxide dismutase (SOD), and glutathione peroxidase (GPX), and reducing malondialdehyde (MDA) and inducible nitric oxide synthase (iNOS), bee pollen decreased cisplatin-induced liver and renal damage in rats [63]. Considering the bioactive compositional profile and the multitude of health benefits offered by bee pollen, it has received unanimous recognition as “the perfectly complete food” and a “functional food.”

Other beehive products, such as bee bread, beeswax, and bee venom, are also reported to possess beneficial health effects. For instance, beneficial bacteria in bee bread strengthen the immune system, while polyphenols and vitamin C protect against free radicals. Bee bread contains vital minerals, proteins, amino acids, fatty acids, co-enzymes, carbohydrates, and essential vitamins, giving it a high nutritional value and classifying it as a functional food [18]. It functions as a detoxifier, accelerates digestion, and improves liver function due to its high antioxidant content, besides lowering the risk of cancer.

Beeswax is known to possess anti-inflammatory and antimicrobial properties. These properties provide the ideal nutritional balance for soothing the skin and keeping it clean and bacteria-free, besides promoting the healing of wounds and fighting infection. Furthermore, beeswax may help decrease cholesterol and protect the stomach from ulcers triggered by non-steroidal anti-inflammatory medicines (NSAIDs) [15].

Melittin, an active chemical found uniquely in bee venom, accounts for around 50% of its dry weight and has been demonstrated in certain research to possess antiviral, antibacterial, and anti-inflammatory properties [64]. Furthermore, bee venom comprises various bioactive compounds, such as peptides and enzymes, that have the potential to be beneficial in the treatment of inflammation and central nervous system illnesses such as Parkinson’s disease, Alzheimer’s disease, and amyotrophic lateral sclerosis [28]. The peptides apamin and adolapin, which are also found in bee venom, have been found to have anti-inflammatory and pain-relieving qualities. Bee venom has been shown to be effective in treating multiple forms of cancer, including ovarian cancer, prostate cancer, and malignant hepatocellular carcinoma. Furthermore, recent research has indicated that bee venom has anti-cancer properties against breast cancer [65].

### 1.3. Sustainability of Honeybee Products and Their Valorization

Beekeeping activity [66], which benefits society in several systems, aids in the development of global sustainability as well as the sustainable development of rural regions [67,68]. It provides substantial advantages for society in terms of both the economy and the environment. Beekeepers profit financially from the use of their products in food and medicine. The productivity of crops is directly impacted by pollinator variety and density [69]. Organic apiculture requires adherence to certain precise principles, while apiary management should be in accordance with the bees’ biological cycle, habits, and ability to provide nutritious food [70]. A beekeeper must adhere to the principles of organic products based on European rules to practice organic beekeeping, and an independent authority conducts yearly apiary inspections.

The essential utilities produced by honey bees and beekeeping are classified into three pillars: environmental, socioeconomic, and sociocultural [71]. Honey bees are marketed as live beekeeping materials to start a new colony or to complement pollination services provided by natural pollinators, and they are employed to pollinate horticulture crops and fruit trees, thereby increasing productivity, quality, profitability, and farmer revenue. Almost 90% of the 107 most significant crop varieties worldwide are known to be visited by honey bees [72]. Intricate ecological systems depend on pollination, which is also crucial to agricultural productivity. European honey bees (*Apis mellifera*) have been introduced to agricultural systems all over the world to increase pollination and fruit output [73]. The most essential function of bees for our biodiversity is pollination. The complex society of *Apis mellifera* is demonstrated by the coexistence of many generations within a colony, collaboration in child care, and division of labor into queens, drones, and workers [74]. Because of its form and behavior, *Apis mellifera* is able to interact with a wide range of plant species, making it a supergeneralist interactor [75]. Besides their active role as pollinators, honey bees (both adults and larvae) are anticipated to serve as a source of insect-derived food in the future. It has been thoroughly investigated as a source of human food. Due to its significantly higher protein and lower fat levels compared to other traditional food sources, it has been discovered to constitute a good source of nutrition [76]. It is one of the most important bee products that is gaining appeal as a functional food due to its high concentration of bioactive substances, including proteins, dietary fibers, lipids, carbohydrates, and minerals, that have been shown to help improve physical as well as mental wellness.

Besides this, honeybees possess properties as bioindicators and passive bio-accumulator organisms, making them excellent agents for monitoring large areas, especially in locations where infrastructure is lacking [77]. Honeybees are simple to grow, nearly widespread in our environment, and have minimal dietary needs. Honeybee colonies undergo fast, continuous renewal as a result of their high rate of reproduction and relatively short average lifespan. These creatures do not store contaminants in their tissues for long periods of time; instead, they transfer the substances they gather to their products, such as honey, which is also employed as a bio-monitoring tool [78]. Honeybees’ bodies are coated with hair, making them ideal for collecting particulate elements encountered during their interactions with the environment. Honeybees are a very effective way of acting as bio-accumulators since they complete several ground surveys each day, during which they bring various materials into the hive. Once within the hive, honeybee products are simple to collect and check for pollutants [78]. Honeybees, on the other hand, are extremely sensitive to the majority of agrochemicals. The presence of certain very poisonous substances (in the case of pesticides, to which they are extremely sensitive) can significantly increase bee mortality, emphasizing the uncontrolled spread of such toxins across the ecosystem. After ingesting less hazardous contaminants, bees may exhibit physiological changes that can be detected through chemical and biochemical studies. The pollutant content of their surroundings is reflected in the number of toxic residues on or inside their bodies and the contamination of beehive products, underscoring their role as effective bio-indicators [78]. However, the viability of present-day and future bee farms is in jeopardy due to several technical and financial issues, including substantial colony losses and very fluctuating honey outputs [79]. Significant honeybee colony losses occur all over the world as a result of the conflict between the use of pesticides and the need for honey bees. To protect the honeybees against pesticide exposure, the following three strategies have been classified: (1) reduced pesticide use and residues in pollen/beebread and nectar; (2) improvement of honeybees’ resistance to pesticides; and (3) interference of the exposure route during pesticide application [80].

With growing awareness of waste management and sustainable development, there has been a keen interest among researchers in reusing products derived from nature. One major discipline with endless scope for utilizing natural products is biomaterial engineering for various biomedical applications. Several studies describe the progressive increase in biomaterials enhanced by honeybee products that have shown antibacterial, regenerative, and immunomodulatory benefits. These characteristics can be applied to a scaffold for biomedical use to modify its mechanical properties, prevent its rapid deterioration, bacterial infection, and cell toxicity, and enable local administration [24]. By subtly regulating the inflammatory response, the application or transplantation of the scaffold functionalized by honeybee products into the body can promote the healing and restoration of damaged tissues [24]. Therefore, beeswax and pollen have been accounted for in several studies as functionalized biomaterials conjugated with gelatin, chitosan, carbon nanotubes, or antibodies to target cancer cells, wound healing, and additives [81,82,83].

## 2. Biomaterial Fabrication and Improvement by Honeybee Products

Presently, the biomedical sector relies heavily on biomaterials to develop solutions to healthcare problems, such as healing from disease or injury and regaining function. As a result of the latest breakthroughs in the field of biomaterial engineering, numerous biomaterials that can interact with biological systems to execute, enhance, replace, or restore the native function of a damaged organ have been produced [84]. They have potentially found applications in multiple aspects of biomedical science, such as wound care, implants, drug delivery, regenerative medicine, tissue engineering, and, more recently, three-dimensional (3D) bioprinting. Briefly, a “biomaterial” is defined as a substance of natural or synthetic origin that has been developed to interact with biological elements, usually intended for a medical purpose, either therapeutic or diagnostic, without triggering an adverse response. Biomaterials include metals, polymers, ceramics, and composites.

Researchers’ interest in developing innovative biomaterials focused mostly on polymer-based materials. Polymer-based biomaterials can be fabricated into different forms, such as films, sponges, hydrogels, electrospun nanofibers, and nanoparticles, with each having its merits and demerits. For novel biomaterial engineering applications, the characteristics of biomaterials are required to be continuously improved. Biomaterial functionalization is crucial in modifying the surface of biomaterials to better adapt them to their physiological microenvironment and provide the necessary clinical outcomes [85]. Biomaterials can be bio-functionalized by a variety of adjunct compounds that are biologically active within the biomaterial matrix, thereby conferring them with specific properties including antimicrobial, anti-inflammatory, anti-cancer, and osteoinductive effects. The inclusion of bioactive elements into biomaterials can be done through various methods such as entrapment, adsorption, electrostatic deposition, surface modification, and internal incorporation [85,86]. In the majority of instances, the incorporation of bioactive substances from natural sources has been a promising and effective strategy to enhance the application potential of biomaterial scaffolds. In this regard, bee-derived products such as honey, propolis, and royal jelly comprise a blend of phytochemicals with a diverse range of reported bioactivities like antibacterial, anti-inflammatory, and antioxidant properties. Likely so, they have received quite some attention for being used as adjunct materials for the functionalization of polymeric biomaterials [24]. For instance, El-Kased et al. fabricated a topical hydrogel using a blend of chitosan and honey that showed antimicrobial properties against bacterial strains causing burn wound infections and demonstrated that the chitosan-honey gel possessed better healing effects [87]. In another study, biodegradable hydrogels were developed by Sharaf et al. using a combination of β-cyclodextrin and κ-carrageenan to deliver propolis extract, which had a direct impact on the antibacterial and anti-fungal properties of hydrogels in a dose-dependent manner [88]. In their study, Khodabakhshi et al. demonstrated that polyurethane foams functionalized with propolis improved in vitro fibroblast migration and promoted in vivo wound healing, besides possessing broad antimicrobial properties [89]. Additionally, polyvinyl alcohol (PVA) scaffolds bio-functionalized by Tavakoli et al. using MH allowed a sustained release of honey into the wound bed which elicited an antibacterial effect against *S*. *aureus* and promoted fibroblast proliferation [90]. Ramirez et al. recently studied the incorporation of extracellular vesicles of royal jelly into a type-I collagen matrix which showed efficacy against the formation of biofilms by *S*. *aureus* as well as promoted fibroblast migration and proliferation, thus acting as a potential candidate for delivery vehicles in wound healing therapies [91]. Methylcellulose-based freeze-dried foams crosslinked with MH fabricated by Schuhladen et al. demonstrated increased hydrophilicity and wettability since MH reduced the contact angle [92]. Furthermore, the incorporation of MH in the biomaterial was found to promote the migration of fibroblasts and keratinocytes as well as show antibacterial activities against *E*. *coli* and *S*. *aureus*. From the literature, it is quite evident that most bio-functionalization of polymeric scaffolds using bee-derived products has been centered around wound dressing applications. This is particularly attributed to the well-reported antimicrobial properties showcased by almost all products derived from the beehive. However, bio-functionalized scaffolds for tissue regeneration applications have also been reported recently by Ahi et al., who developed composite films using polylactic acid (PLA), polycaprolactone (PCL), and propolis intended for periodontal-guided tissue regeneration [93]. The recent developments in the use of beehive products for biomaterial functionalization biomedical applications have recently been reviewed extensively [24].

Even though multiple studies are reported in the literature involving the use of standard beehive products (e.g., honey, royal jelly, and propolis) for developing functionalized biomaterial scaffolds, the incorporation of bee pollen as an adjunct material remains poorly investigated. Likely so, there are currently no existing reviews that summarize the existing knowledge on this topic. Even a former review of ours, which comprehensively reviewed the outcomes of using beehive products for biomaterial development, did not focus on bee pollen as a potential target for developing scaffolds for wound healing and other biomedical applications. This review aims to collect the existing literature involving bee pollen used in biomaterial development to fill the knowledge gap regarding the suitability of bee pollen as a potential material for biomaterial functionalization. We have highlighted the major bioactive components and reported the health benefits of bee pollen, which have provoked researchers to consider its applicability in biomedical applications by improving a biomaterial’s properties. Additionally, recent studies reported in the literature where bee pollen incorporation has improved a biomaterial’s properties, allowing its use in biomedical applications, or where the outcomes reported can be translated towards biomedical applications, have been reviewed. Moreover, we have also stressed the need for future research regarding the use of bee pollen in biomaterial functionalization, besides commenting on the possible future scope of such functionalized biomaterials in the development of tissue-engineered grafts for pre-clinical models and in the in vitro culture of stem cells for regenerative medicine applications.

## 3. Current Landscape of Functionalized Biomaterial Development Using Bee Pollen

Biomaterials interact with human cells, tissues, or organs and, in some cases, perform their functions or provide a therapeutic outcome. Biomaterials are used to improve human health and quality of life by functionally restoring different tissues [94]. The nature and original intent of biomaterial research have been to provide solutions, insights into host-material responses, and medical products to address unmet medical and clinical needs and scientific questions. Consequently, biomaterial research has grown tremendously and developed into a multidisciplinary and cross-functional field [95].

A key consideration during biomaterial development is to realize the end application of the biomaterial being developed. Based on this, there are several criteria that a biomaterial needs to satisfy in terms of its biocompatibility, biodegradability, physical properties, and chemical properties. An elaborate description of these factors does not fall under the scope of this review; hence, it is not discussed ahead. This review will focus on biomaterial development using polymeric materials since they have emerged as the cornerstones of biomedical applications and represent the most versatile and thoroughly researched biomaterials. In this context, both natural polymers (alginate, agarose, carrageenan, cellulose, chitosan, collagen, fibrin, gelatin, hyaluronic acid, keratin, and silk) as well as synthetic polymers (polyacrylic acid (PAA), polylactic acid (PLA), polyvinyl alcohol (PVA), polycaprolactone (PCL), and polylactic-co-glycolic acid (PLGA)) have been used alone or in combination for developing biomaterial scaffolds for various biomedical applications [96]. Natural and synthetic polymers can be prepared in different forms, e.g., films, electrospun fibers, sponges, hydrogels, 3D-printed scaffolds, and nanoparticles [96,97]. These polymeric biomaterial scaffolds can be bio-functionalized by loading natural products comprising bioactive molecules into them to improve their properties and functions. Beehive products represent one such emblematic natural source of various bioactive molecules that have been used for improving biomaterial properties through functionalization. These natural products are rich in bioactive compounds, which together bestow numerous bioactivities on these byproducts, such as antimicrobial, antioxidant, and anti-inflammatory properties [98].

Bee pollen, even after its reported bioactivities and health benefits, remains an underutilized biomaterial functionalization material to date. Incorporating beehive products such as bee pollen may cause cytotoxicity to cells and tissues [24]. However, using a biomaterial scaffold can effectively enable the release of a controlled, safe dose of the bioactive compounds in bee pollen to elicit desirable functionalities in the resulting scaffold. An essential aspect of incorporating bioactive natural substances or their extracts within polymeric biomaterial scaffolds lies in carrying out an extensive investigation to determine their appropriate dosage concentration within a biomaterial scaffold. This value may vary depending on the polymer used and the target application in order to achieve the most beneficial effects both in terms of biocompatibility and functional bioactivities such as cell infiltration and proliferation, modulation of inflammation and ROS, infection prevention, and tissue regeneration while maintaining ideal biomechanical characteristics [24].

In general, beekeepers harvest pollen from bees using pollen traps, which fit over the entrance to a hive and have apertures just large enough for a returning forager to squeeze through. The pollen carried on the bee’s rear legs is knocked off during the squeezing operation and slides through a screen into a drawer where it can be gathered by the beekeeper. Because fresh bee pollen is highly perishable, pollen captured in a trap must be collected daily and quickly kept in a dehumidified environment to prevent mold growth and preserve the pollen’s nutritional and therapeutic characteristics, which are highly desirable during biomaterial functionalization [99]. Fresh pollen typically includes 10–12% water, whereas dry pollen contains roughly 4% moisture. It is believed that drying bee pollen in the sun can reduce pollen potency by up to 50% due to antioxidant oxidation. As a result, freezing pollen soon after harvesting is the best approach to preserving it, with refrigeration being the next best option. When drying pollen, it is ideal to dry it at a temperature of roughly 86 °F (30 °C) and in the dark to maintain its bioactive contents. Ensuring the preservation of bee pollen’s bioactive properties during its collection and processing is critically important when utilized during biomaterial fabrication. It is widely acknowledged that bee pollen can be affected by pesticides, heavy metals, metalloids, and mycotoxin-producing molds in the environment. Furthermore, pollen from certain plant species initially contains rather high quantities of hepatotoxic pyrrolizidine alkaloids. These items may also contain allergens and pollen grains from genetically engineered plants [100]. Although pesticide residues do not normally pose a long-term risk to biological applications, the predicted acute exposure levels can be near the acute reference dose (ARfD). Because the arsenic, cadmium, lead, and pyrrolizidine alkaloid content of bee pollen may pose health risks, it is recommended that a maximum limit for these substances be established, as well as extensive monitoring of their concentration and quality analysis of the bee pollen to be performed prior to using them as a functionalization agent [101].

The studies reporting the combined use of bee pollen and different types of fabricated scaffolds have been summarized in Table 2; the same studies are discussed further below. Owing to the close compositional profile and bioactive properties between botanical pollen and bee pollen, we review the studies involving natural pollen, which can provide similar or better outcomes compared to the use of bee pollen.

### 3.1. Bee Pollen for Natural Polymers

Natural polymers, particularly polysaccharides, have been used for developing functionalized biomaterials using bee pollen. Polysaccharides have long been regarded as advantageous for biomaterial development due to their wide availability, biocompatibility, hydrophilicity, immunoactivity, and ease of chemical modifiability [112]. Owing to their partial similarity with the extracellular matrix (ECM) of humans in terms of composition and properties, polysaccharides have received a lot of attention as promising materials for developing scaffolds intended for various biomedical applications. Moreover, these polymers undergo biodegradation within physiological systems without forming any toxic by-products; hence, they do not elicit cytotoxic effects on the cells within which they are introduced. Additionally, by simply modifying the functional groups on the surface of polysaccharide molecules, definite properties such as tunable mechanical characteristics and controllable tissue responses can be achieved with the resulting biomaterial. Consequently, polysaccharides have frequently been used as practical building blocks in the development of novel biomaterials for wound healing dressings, drug delivery systems, cell-encapsulating biomaterials, and scaffolds for tissue engineering [113].

**Figure 2:**
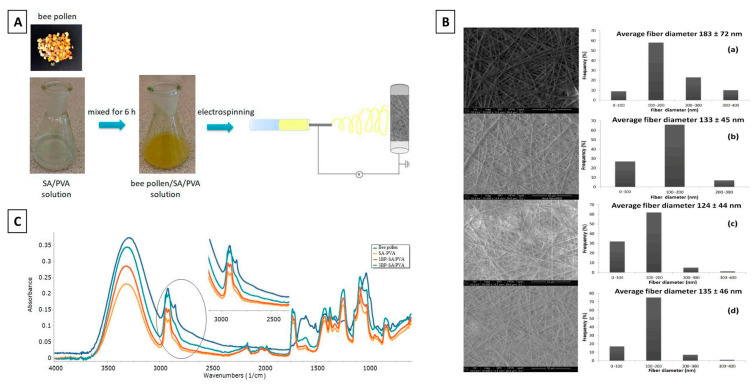
Illustration of bee pollen incorporated SA/PVA electrospun nanofibrous mats. (**A**) Schematic of the preparation of bee pollen-loaded SA/PVA electrospun nanofibrous mats by electrospinning. (**B**) SEM images and fiber diameter distributions of the electrospun SA/PVA fibers obtained by blending different fractions of 1%, 2%, and 3% of bee pollen in an electrospinning solution. (**C**) ATR-FTIR spectra of the bee pollen, the electrospun SA/PVA, and bee pollen-loaded SA/PVA nanofibers. Reproduced with permission from [102], Creative Commons Attribution (CC BY), 2021.

Alginate, which is an anionic polysaccharide derived from the cell walls of brown algae, is among the most widely available polysaccharides in the world alongside cellulose and chitosan. Commercially available alginate is usually extracted as its water-insoluble form, alginic acid, and thereafter converted to soluble and purified sodium alginate [114]. Alginate-based biomaterials are among the best-studied biopolymers widely used for biomedical engineering applications due to their desirable characteristics, which include a better therapeutic payload, targeted efficiency, pH responsiveness, biocompatibility, low toxicity, structural similarities to the extracellular matrix, gelling abilities, wide availability and low cost [115,116]. Due to their gelling properties in the presence of divalent cations, alginate-based scaffolds are regarded as excellent matrices for the encapsulation of cells and bioactive agents such as bee products and extracts, [117]. Although most studies have reported the use of alginate in the development of hydrogels, another popular technique known as electrospinning has been utilized to prepare electrospun alginate-based nanofibers which have found use in biomedical applications such as wound dressing [118,119], tissue engineering [120,121,122], and drug delivery systems [123]. Electrospun fibers have the ability to mimic the morphology of natural sub-micron fibers found in human tissues. Additionally, electrospun nanofibers have a high surface area-to-volume ratio, which helps explain their popularity [124]. Nevertheless, the incorporation of bee pollen into alginate scaffolds was poorly investigated until recently when, Pakolpakçıl, et al. developed bee pollen-loaded sodium alginate and polyvinyl alcohol (SA/PVA) nanofibrous mats through an environmentally sustainable green electrospinning technique [102]. The preparation and characterization of these functionalized electrospun nanofibrous mats are briefly depicted in Figure 2. The inclusion of bee pollen in the SA/PVA solution increased the apparent viscosity and electrical conductivity of the solution, facilitating the electrospinning process. This was evident from Scanning Electron Microscopy (SEM) revealing nanoscale features on the resulting electrospun nanofiber mats with an average fiber diameter of 100–150 nm. The FTIR spectrum confirmed the incorporation of bee pollen into the SA/PVA nanofiber mats as observed by characteristic peaks of bee pollen at 3300 cm^−1^, 2922 cm^−1^, 1606 cm^−1^, 1514 cm^−1^, and 1028 cm^−1^, assigned to OH groups, CH stretching, CC stretching, and CO stretching, respectively, [125]. Interestingly, an increase in the glass transition temperature (T_g_) was ascertained in the SA/PVA nanofibrous mats in the presence of bee pollen, suggesting that the presence of bee pollen improved the ordered association of the SA/PVA molecules via hydrogen bond formation. Because bee pollen is believed to possess antimicrobial, antioxidant, anti-inflammatory, immunostimulatory, and local analgesic properties, it is plausible that the bee pollen-loaded SA/PVA mats may have the potential to be translated into several biomedical applications, particularly as antimicrobial wound dressings.

Another popular polysaccharide frequently used in biomaterial development is the deacetylated product of chitin, chitosan. The applicability of chitin in biomaterial fabrication is limited by its poor solubility in water and most organic solvents. Instead, chitosan, which is a linear polysaccharide derived from partial chitin deacetylation, reportedly has remarkable properties that account for its suitability in biomaterial fabrication for different biomedical applications [126]. The presence of amino groups (-NH2-) in the chitosan structure, which undergoes protonation and subsequently offers solubility in aqueous solutions of dilute acids, accounts for several remarkable properties of chitosan, paving the way for unique opportunities for the development of functional biomaterials. Besides the polycationic nature of chitosan, its biocompatibility, biodegradability, and non-toxicity make its use suitable for biomedical applications such as hemostatis, wound healing, and tissue engineering scaffolds [127].

For instance, chitosan films with antimicrobial and antioxidant properties were prepared by Baysal et al. by encapsulating varying amounts of bee pollen and apple cider vinegar inside the polymer matrix [103]. The presence of bee pollen and apple cider vinegar within the films was confirmed through FTIR. Interestingly, the total polyphenol content of the freeze-dried chitosan films, calculated as gallic acid equivalent (GAE), increased with the incorporation of bee pollen and apple cider vinegar, with a higher value being observed in the group receiving a greater amount of bee pollen (CSy) as compared to apple cider vinegar (CSx). These results were consistent with the results of a DPPH assay carried out to evaluate the antioxidant potential of the composite films, which revealed a higher antioxidative potential of the films containing a higher amount of bee pollen extract. Most importantly, investigation of the antibacterial properties of the composite films against *Listeria monocytogenes*, *Salmonella sp.*, *Escherichia coli*, and *Staphylococcus aureus* using the good diffusion method revealed the highest antimicrobial activity in the CSy group, with the zone of inhibition being observed for all the bacterial species. Such excellent antioxidant potential and the antimicrobial properties of the films, particularly against *Escherichia coli* [128], and *Staphylococcus aureus* [129], which are known to cause infections on wound sites, mandate the translation of this work into the biomedical sector towards developing wound dressings.

Due to the structural properties of chitosan, it can be easily modified and fabricated into different forms to enhance its biological and chemical properties. Hydrogels are among the most widely used forms of chitosan-based scaffolds, offering multiple benefits in ease of fabrication, cytocompatibility, and highly tunable properties, allowing their use in various biomedical applications. In another study, Tyliszczak et al. prepared hydrogels using gelatin and beetosan^®^, a special form of chitosan obtained through the multi-step processing of dead bees [104]. In this study, the authors functionalized the prepared hydrogels using bee pollen due to their inherent bioactive properties. SEM analysis of the prepared bee pollen-modified hydrogels revealed a more homogeneous surface than their unmodified counterparts. Furthermore, wettability studies to assess the hydrophilicity of the hydrogels revealed that hydrogels modified with bee pollen had a significantly lower contact angle than the unmodified hydrogels. The authors studied the cytotoxicity of the prepared hydrogels using an MTT assay on two different cell types. Interestingly, the bee pollen-modified hydrogels exhibited anti-cancer activities as observed by a high number of non-viable Jurkat cells (of cancerous origin) after 7 days of exposure to the modified hydrogels, therefore holding immense potential in biomaterial development for cancer therapy. On the contrary, the fibroblast cell line WEHI 164 did not show any detrimental signs upon exposure to the bee pollen-functionalized hydrogels. This underscores the potential of these hydrogels in biomedical applications such as wound healing and skin tissue engineering, where fibroblasts are known to play an important role.

Towards this end, the positive role of bee pollen in wound healing was investigated by Bacha et al. [105]. This was achieved by fabricating hydrogels using sodium carboxymethyl cellulose (CMC) as a biopolymer and incorporating bee pollen. CMC, which is one of the most commonly modified forms of cellulose, the most abundant polysaccharide in the world, has been identified as a promising biomaterial due to its chemical properties, which enable further modification for a wide range of biomedical applications [130]. By simply controlling the surface chemistry of CMC, the obtained scaffolds can be made to showcase properties such as mechanical strength and resistance to in-vivo breakdown, as well as biocompatibility and providing reactive surfaces for protein binding. These benefits were considered while preparing bee pollen-loaded CMC hydrogels, which were subsequently treated on rabbits inflicted with burn wounds using a cylindrical stainless-steel rod heated to 100 °C. Following 24 h of treatment with bee pollen-loaded CMC hydrogels, the rabbits were found to be healthy, with no signs of skin irritation or edema. This served as the foundation for deeming the hydrogels safe for dermal applications, and they were hence studied for their possible role in promoting wound healing via monitoring the extent of wound closure. The treatment groups receiving bee pollen-loaded CMC hydrogels reportedly showed superior healing outcomes without any complications, with approximately 95% wound closure achieved after 25 days. The findings of this study corroborate the idea that bee pollen-modified biomaterials can encourage wound healing by lowering oxidative stress in the wound, inhibiting inflammation, and inducing collagen synthesis. This study’s findings confirmed that bee pollen-loaded CMC hydrogels could be used as topical burn healing dressings.

Although most studies involving the fabrication of bee pollen-functionalized scaffolds reported improvements in antioxidant and antimicrobial properties suitable for wound healing or food packaging applications, attempts have also been made to explore other aspects that can be improved by adding bee pollen to biomaterial scaffolds. In line with such attempts, Macieira et al. developed bee pollen-incorporated biodegradable films using starch via a two-step process involving solvent casting and its subsequent evaporation [106]. It was observed that the incorporation of pollen into starch resulted in the formation of heterogeneous films with moderate to light permeability. Interestingly, the incorporation of bee pollen in different proportions reportedly improved the thermal stability of films with higher bee pollen content, as seen through limited weight loss at 100 °C during thermogravimetric analysis. This could be attributed to the high intermolecular bonding of bee pollen with water molecules. These results were in line with differential scanning colorimetry analysis, which showed differences in the curves obtained by varying the pollen content in the films. Additionally, the interaction between bee pollen and starch significantly improved the mechanical properties of the films in terms of elongation capacity but not their tensile properties. A biomaterial can be used to aid in the healing process, replace a defective part through tissue engineering, or serve as a drug delivery vehicle. Thus, it could remain temporarily or permanently in the body, depending on the application [131]. The thermal stability of biomaterials can significantly impact their performance post-in vivo implantation or when interfacing with the biological environment. Similarly, appreciable mechanical properties can be highly desirable in hard tissue engineering. Hence, this study reported that improving mechanical and thermal characteristics in polymer films holds immense potential for expanding the scope of bee pollen-modified materials for translation into various biomedical applications.

### 3.2. Bee Pollen for Synthetic Polymers

Even though natural polymers have been considered promising building blocks for biomaterial development using bee pollen, the use of synthesized polymers for this purpose has only been realized recently. Because of their numerous advantages over their natural polymer counterparts, synthetic polymers have demonstrated significant potential for fabricating biomaterial scaffolds. Their mechanical properties are more diverse than those of natural polymers; hence, they are frequently used for reinforcing the mechanical properties of natural polymer-based scaffolds [132,133]. Synthetic polymers have process-controllable batch-to-batch consistency, well-defined chemistry, and highly tunable properties. Together, these properties facilitate large-scale production, ensuring quality consistency in the biomedical field [134]. Additionally, these polymers can be functionalized with natural polymers through chemical modifications or the addition of bioactive components to impart new properties to the resulting scaffold [135]. For instance, Tyliszczak et al. modified PAA hydrogels with bee pollen to study whether the addition of a bioactive component improved the properties of the resulting scaffold [107], used in wound dressing application. SEM micrographs revealed bee pollen to be encapsulated almost entirely within the polymer matrix. Although the swelling potential of the modified hydrogels was altered by the incorporation of bee pollen, which reduced the available volume within the polymer matrix, the resulting bee pollen-PAA hydrogel demonstrated a fairly high sorption capacity against various liquids commonly used in biomedical testing, such as Ringer’s solution, simulated body fluid (SBF), and pseudo-extracellular fluid (PECF). The results of a series of incubation experiments demonstrated that these materials are stable in body-simulated conditions, in addition to circumstances mimicking wound environments, over a long period of time. Given that PAA hydrogels have already been used as wound dressing materials, this research holds promise for the development of functionalized dressings that are expected to have antioxidant, anti-inflammatory, and antimicrobial properties, which can expedite healing in chronic wounds that are characterized by delayed healing due to multiple complications such as oxidative stress and inflammation [136].

### 3.3. Bee Pollen for Tissue Engineering

Available literature strongly suggests that the use of bee pollen-modified biomaterials has been more inclined toward applications involving the skin. However, recent research has been focused on deciphering the role of bee pollen in other tissues [137]. Some in vitro studies suggest that bee pollen has a stimulatory effect on bone formation and an inhibitory effect on bone resorption; however, in vivo studies to substantiate these findings have been limited. There were contrasting reports based on in vivo findings regarding the effect of bee pollen on bone development. For example, Tomaszewska et al. highlighted that bee pollen supplementation had an adverse impact on the mechanical resilience of the tibia but had a beneficial impact on trabecular bone homeostasis [138]. Another study by Yamaguchi et al. showed that the dietary intake of water-solubilized extracts of bee pollen of *Cistus ladaniferus* by diabetic rats prevented bone loss due to diabetes, observing increased alkaline phosphatase (ALP) levels, inhibition of resorption levels, and osteoclast cell formation [139]. Despite the growing number of research publications investigating the effect of pollen on bone, their role in developing scaffolds for tissue engineering remained poorly explored until a very recent study by Zakhireh et al. [108]. The researchers explored a bottom-up tissue engineering approach involving hollow pollen grains as scaffolding building blocks for engineering bone tissue using human adipose-derived mesenchymal stem cells (hAD-MSCs) [108]. Spherical bead-shaped pollen grains offering a high surface area-volume ratio were isolated from *Pistacia vera* L. and processed via mild alkaline treatment to obtain hollow pollen grains (HPGs). These HPGs were subsequently used to encapsulate bone morphogenetic protein 4 (BMP4), which is known to induce osteogenic differentiation. Field emission SEM analysis revealed that the HPGs-BMP4 building was able to interact with the hAD-MSCs, thereby providing better cell adhesion sites. The investigation of apoptotic gene expression revealed a lower BAX/BCL2 ratio, indicating that HPGs protect hAD-MSCs from losing viability. The osteoconductive potential of the HPGs-BMP4 building blocks was demonstrated by increased ALP activity and expression of osteogenic genes, which are RUNX Family Transcription Factor 2 (RUNX2) and osteocalcin, and a synergistic effect on osteoblast maturation. Overall, the use of HPG as a scaffolding building block resulted in increased cell adhesion, viability, and confluence due to several factors, including a high surface-to-volume ratio, surface functionality, homobrochate ornamentation, and the potent antioxidant activities of the pollen, attributed to high levels of phenolic compounds such as flavonoids. Pollen grains directly isolated from the flower are known to have a similar compositional profile to bee pollen, as worker bees collect the natural pollen from plant anthers and mix it with minimal saliva or nectar before transporting it to the hive. As a result, the bioactive properties possessed by normal pollen do not change appreciably when converted to bee pollen. Therefore, this study forms the basis for subsequent research toward developing bee pollen-functionalized scaffolds for bone tissue engineering.

### 3.4. Bee Pollen for Bioink Development

**Figure 3:**
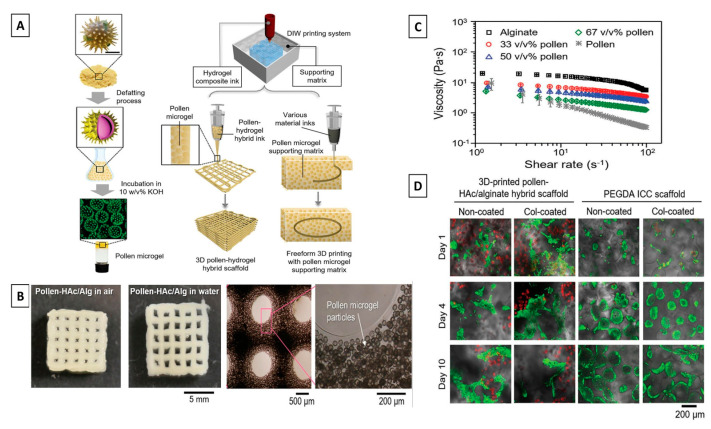
Pollen-derived microgel suspension for bioink development. (**A**) Schematic depiction of pollen microgel as a bioink and supporting matrix material for 3D bioprinting applications. (**B**) Optical images of hyaluronic acid/alginate scaffolds. (**C**) Shear thinning behavior of pollen-polymer blends prepared using different volume ratios of polymer-to-pollen. (**D**) Live/dead staining of Huh-7.5 cells cultured on 3D-printed pollen–hyaluronic acid/alginate hybrid scaffold (noncoated, Collagen-coated) compared with PEGDA ICCs (noncoated, Collagen-coated) on days 1, 4, and 10. Live cells were stained with calcein-AM, fluorescein green, whereas dead cells were stained with EthD-1, fluorescein red. Reproduced with permission from [109], John Wiley and Sons, 2021.

A paradigm shift involving the integration of 3D printing technology into traditional tissue engineering approaches has recently revolutionized biomedical research for developing such tissue substitutes [140]. By using a layer-by-layer approach, the resulting technology, known as 3D-bioprinting, has shown great potential in tissue fabrication with structural control from micro- to macro-scale [141]. The benchmark for 3D-bioprinted constructs is to provide a biomimetic structural microenvironment that facilitates tissue formation and promotes host tissue integration. Bioinks, which are defined as biopolymer solutions of a single or a combination of biopolymers in hydrogel form encapsulating the desired cell types used to create tissue constructs during the bioprinting process, are an important topic of research. In order to improve their properties, bioinks have frequently been blended with various bioactive components, including some originating from the beehive [142,143,144]. Recently, Chen et al. reported an engineered sunflower pollen-derived microgel suspension obtained by an environmentally friendly method involving the transformation of pollen grains into stimulus-responsive microgel particles as shown in Figure 3 [109]. The engineered pollen-derived microgel suspension was able to serve as a functional reinforcement for alginate/hyaluronic acid composite hydrogel bioinks. The pollen microgels demonstrated a number of exceptional benefits as bioink components. Irrespective of the amount of pollen microgel particles present, the pollen microgel-hydrogel composite ink demonstrated satisfactory printability. In the hydrogel matrix, the microgels were evenly distributed and did not significantly aggregate, producing printed structures that were uniform and had good structural integrity. The pollen microgel particles improved the mechanical and physiological stability of alginate/hyaluronic acid hydrogel scaffolds. In order to demonstrate the potential of pollen microgel-alginate/hyaluronic acid scaffolds for 3D cell culture applications, the researchers seeded their scaffolds with Huh-7 cells, which demonstrated 94% cell viability. All these observations taken together warrant extensive research involving bee pollen as an active component of novel bioink for future bioprinting applications.

### 3.5. Bee Pollen for Nanoparticle Development

Innumerable advancements have been made in the biomedical pharmacology sector to address previously incurable diseases through the development of new drugs and drug delivery platforms. Drug delivery systems are becoming more advanced, with an emphasis on a better controlled release profile, maintaining therapeutic efficacy, targeting specific modes of action for the active ingredient, and avoiding the systemic release of the active substance in an effort to reduce the risks and drawbacks associated with conventional administration routes. In this regard, nanotechnology is becoming increasingly important because it may be able to address some of the problems related to the aforementioned conventional administration routes [97]. Nanotechnology, in particular how drugs are formulated and delivered, has been revolutionized by the clever use of nanoparticles, which have been developed to mimic or change biological processes through the application of nanotechnology [145]. By increasing their solubility or ease at which they can cross biological membranes, nanoparticles could be optimized to increase the drug’s bioavailability, while by varying the composition of the nanoparticulate system, drug release could also be regulated and maintained at therapeutic levels. Bee pollen has inherent anti-cancer properties, which have been researched [146]. Previously, bee pollen incorporated within biomaterial scaffolds has been reported to exert cytotoxic effects on Jurkat cells [104]. Additionally, cisplatin, a chemotherapeutic drug, when supplemented with bee pollen methanolic extracts, demonstrated improved anti-proliferative activity on the breast cancer cell line MCF-7 [147]. A recent study by Hanafy et al. reported the development of bee pollen extract (BPE) based polymeric nanoparticles (BPENP) using bovine serum albumin (BSA) [110]. The nanoparticles were coated with folic acid-linked protamine and subsequently targeted A549 lung cancer cells. The particle size of fabricated nanoparticles varied between 25 and 40 nm on average. BPE and its NPs demonstrated strong anti-cancer effectiveness on A549 lung cancer cell lines in a dose-dependent manner. The concentration of inhibition (IC50) determined from the treatments was lower after 48 h than after 24 h in all treatment groups. This is strongly attributed to the high flavonoid and polyphenol content of the extract obtained from bee pollen. Furthermore, the combination therapy assay of BPENPs and the chemotherapeutic drug Avastin at their IC50 levels demonstrated a unique synergism, highlighting the therapeutic aspect of these polymeric nanoparticles for cancer therapy by blocking cancer-promoting cellular signaling and activating apoptotic pathways in cancer cells. Another study by Hanafy et al. reported similar findings where administration of BPE encapsulated within hybrid hydrogel nanoparticles significantly improved the anti-cancer effectiveness of Bevacizumab against A549 and MCF-7 cell lines when used in combination [111]. Such findings are a strong impetus for using bee pollen nanoparticles in conjunction with chemotherapy to increase efficacy while reducing the required dose.

### 3.6. Short Summary

Briefly, the past decade has witnessed an increase in the use of bee pollen for developing biomaterials. The varying quality and amount of phenolics contribute significantly to a wide range of biological effects, including antibacterial and antioxidant activity in bee pollen from various sources. There are a lot of extrinsic factors that affect the chemical composition of bee pollen, such as geographical origin, plant source, climatic conditions, race, and the activities of bees [25,148]. During the late winters and early springs, the outdoor temperature begins to increase, thus leading to the blooming of numerous spring flowers. The honey bees become hyperactive and start “foraging”, which means collecting the nectar and pollen from the newly bloomed flowers. While foraging, the bees come into contact with different flowers, and pollen from a variety of plant species sticks to their feet, resulting in the occurrence of multiflora or polyfloral bee pollen. Under certain circumstances, the pollen from a particular species dominates over that of other species due to the fact that unifloral or monofloral bee pollen occurs. Some studies have reported that these bee pollen contain a wide range of macronutrients such as proteins, lipids, and dietary fibers, as well as a variety of bioactive micronutrients like vitamins, minerals, and phenolic compounds. These compositions widely vary depending on the geographical and climatic conditions as well as the plant species [149]. In monofloral bee pollen from various nations, Thakur et al. and De-Melo et al. found an extensive variety of lipid contents: the total lipid contents of *Brassica napus* bee pollen were found to be 12.38%, 7.76%, 7.4%, and 6.6% in India, Greece, Brazil, and China, respectively, while *Cistus* bee pollen from Spain, Greece, and Italy had total lipid contents of 7.2%, 3.80%, and 1.9% [13,150]. In an experiment by Taha et al., the amino acid composition and protein content varied among different floral species in Saudi Arabia. It was seen that date palm and alfalfa bee pollen exhibited high crude protein content and concentrations of amino acids like valine, proline, isoleucine, leucine, phenylalanine, arginine, lysine, cysteine, and metheonine, while bee pollen from sunflower showed low concentrations of these components. Sunflower bee pollen mainly contains high concentrations of amino acids like serine and glutamic acid [151]. Apart from geographical conditions, variations in bee species also play an important role in the presence of some crucial bioactive components. For instance, high quantities of mannitol, a polyol, were present in high concentrations in the bee pollen collected by stingless bee species such as *Tetragonula biroi Friese* from the Philippines, *Melipona subnitida* from Janda, Brazil, and *Trigona* from Malaysia [152,153]. Hence, the authors concluded that the floral origin did not certainly have an impact on the production of mannitol. Rather, it was because of the capability of the stingless bees to convert glucose and fructose into mannitol by their salivary enzymes. Bee pollen constitutes almost all the vitamins with an average content of 0.02–0.7% that are essential for survival, and hence they are also known as “vitamin bombs.” These vitamins vividly vary from species to species as well as by geographical origin [154,155]. The phenolic contents widely varied in pollen collected from different plant species. In a recent study by Barbieri et al., the phenolic content was estimated as Folin-Ciocalteu (FC) reducing capacity and was found to be the highest in the species *Viburnum* (99%) (20.15 ± 0.15 mg GAE/g fw), followed by *Eucalyptus* (19.63 ± 2.53 mg GAE/g fw), 18.98 ± 1.36 mg GAE/g fw in *Prunus*, and 17.82 ± 1.68 mg GAE/g fw in *Brassicaceae*, while the lowest level of 5.78 ± 0.87 mg GAE/g fw was seen in *Viburnum* (96%) [156]. A similar trend was seen in flavonoids, where *Viburnum* (99%) showed the highest level of flavonoid content, followed by *Prunus* and *Eucalyptus*. The lowest level was shown by *Asteraceae*. T. Gabriele et al. demonstrated that the phenolic content of a Tuscan polyfloral bee pollen was similar to that of the above-mentioned species, but the flavonoid contents were relatively higher [157]. Thus, it can be concluded that variations in the environment and species can have a certain impact on the chemical composition and bioactive components of bee pollen as well as other honey bee products. A moderate temperature and tropical countries have more variety of vegetation, which increases the foraging activities of the honey bees, thus resulting in abundant availability of honey bee products that can be utilized for the betterment of mankind.

**Figure 4:**
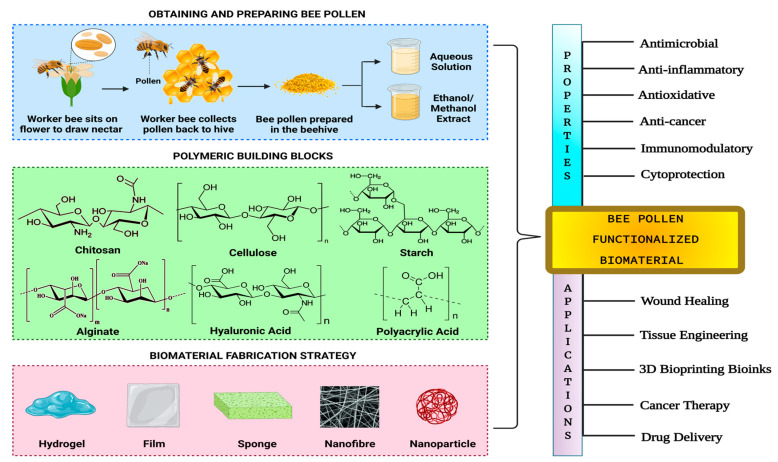
Biomaterial development and functionalization using bee pollen and its properties and applications.

In short, slight variations in the composition of bee pollen can be attributed to differences in foraging area, environmental factors such as soil type, seasonal and regional conditions, beekeeping activities, and bee pollen harvesting methods, but major variations in the composition and nutritional value of bee pollen can be attributed to different botanical origins. Overall, this broad spectrum of bioactive potential reported in multiple forms of bee pollen could be used to create customized systems that detect and eliminate microbial infection or to reduce the level of oxidative stress after the implantation of tissue-engineered structures. Additionally, there is immense promise for the utilization of bee pollen in other aspects of biomedical engineering, such as wound care and drug delivery. Bee pollen obtained from the beehive can either be used in its pristine condition or its extracts may be prepared via an intermediate ethanol/methanol extraction step and subsequently converted into polymeric biomaterial building blocks of different forms to fabricate functionalized biomaterials for different applications (Figure 4). The use of such a naturally available source of bioactive components holds the key to exploring many unsolved areas of biomedical research, besides improving the ongoing ones.

## 4. Discussion

It is safe to mention that research involving bee pollen for biomaterial development is at a very nascent stage, with only a handful of studies available online, among which a few report findings that can be translated into specialized biomedical applications. Nevertheless, the antimicrobial, anti-inflammatory, antioxidant, and immunomodulatory properties of bee pollen and honeybee products have made them highly desirable sources of naturally derived adjunct material for fabricating functionalized biomaterials. Most of the biomaterial development studies using bee pollen have reported antioxidant and antimicrobial properties in the resulting biomaterial. Likely so, the incorporation of bee pollen holds immense promise in the development of biomaterials intended for wound care applications, tissue regeneration, and therapeutics, where such bioactivities would be highly desirable. Wound formation results in the production of abnormal levels of free radicals that can trigger long-lasting inflammation. Recently, the role of biomaterials incorporated with antioxidative properties to scavenge free radicals generated in wound environments and promote wound healing and tissue regeneration has been reviewed by Fadilah et al. [158]. Due to the antioxidant and anti-inflammatory properties of bee pollen, it can help scavenge reactive oxygen species (ROS), preventing cell apoptosis due to excessive free radicals. Such features would be particularly beneficial in tissue engineering applications as well, since biomaterial scaffolds loaded with cells that are implanted in vivo would be shielded from the detrimental effects of free radicals and inflammation evoked by the surgical procedure.

Bee pollen has the highest levels of the amino acids leucine, glutamic acid, valine, isoleucine, threonine, and glycine, along with polysaccharides, vitamins, and minerals [98]. The concentrations of essential amino acids in bee pollen strongly vary with the pollen’s botanical origin [151]. The proteins found in bee pollen can provide cell attachment cues for the cells to adhere to. Cell adhesion to biomaterial scaffolds through the expression of cell adhesion molecules (e.g., integrins) on their surface is an essential event for regulating a wide range of biological functions, including survival, migration, integration, and retention within the scaffold, as well as cellular differentiation and mechanotransduction. Additionally, the polysaccharides present in bee pollen can provide energy and support for cell proliferation during in vitro culture for tissue engineering applications. Vitamins and minerals in bee pollen can also provide essential nutrients for cell growth and differentiation in tissue engineering applications. The antioxidant effects of bee pollen can also help protect cells from oxidative stress, which can further enhance their viability and promote cell adhesion, proliferation, and differentiation. Overall, bee pollen has a promising future in hepatic and cardiac tissue engineering applications, besides bone tissue engineering, as discussed earlier. By combining bee pollen with polymeric materials used as biomaterial building blocks, it is possible to create a biocompatible and bioactive scaffold that can be used to aid in the growth and repair of several tissues [159].

## 5. Future Perspectives

Gradually, there has been growing evidence highlighting the potential of bee pollen to be used in tissue engineering and regenerative medicine applications since it has demonstrated protective functions in certain cell types. The hepatoprotective properties of bee pollen were reported recently, wherein bee pollen was found to protect hepatocytes from oxidative stress and promote the healing of liver damage [160]. This would serve as the foundation to formulate bee pollen-incorporated biomaterials for culturing and encapsulating hepatocytes for engineering liver substitutes or fabricating cell delivery platforms to ameliorate hepatocellular pathologies like cirrhosis. It has been noticed that bee pollen possesses cardioprotective properties, though the precise underlying mechanisms remain unclear. However, it is suggested that bee pollen exerts its anti-cardiomyocyte injury effects by reducing oxidative stress levels and inhibiting inflammatory responses and apoptosis. Of late, Han et al. reported that lotus bee pollen extracts prevented isoproterenol-induced cardiomyocyte hypertrophy in rat H9C2 cell lines [161]. Furthermore, Shen et al. also demonstrated that extracts of *Schisandra chinensis* bee pollen have antioxidative and protective effects against acute myocardial infarction induced by isoprenaline in rats. Therefore, bee pollen-functionalized biomaterials could pave the way for breakthroughs in biomedical science through the development of tissue-engineered engineered/cardiomyocyte delivery solutions for treating and preventing cardiac damage [162].

The clinical applications of biomaterial scaffold systems have been significantly constrained by biomaterial-associated microbial contamination in biologically conducive 3D tissue-engineered constructs [163,164]. This is primarily because of their intrinsic ability to serve as highly suitable cultural systems that provide adequate nourishment. Infections associated with tissue-engineered grafts are brought on by microorganisms that build biofilms and stick to them. This potentially hinders the implantation procedure of tissue-engineered grafts [163]. Even in the absence of biomaterial-associated infection during the pre-implantation period, the successful implantation of biomaterials into physiological environments usually involves an operation requiring the generation of surgical wounds. Here, the risk of post-operative infection causing graft rejection also exists. Antimicrobial biomaterials are rapidly emerging in an attempt to prevent the onset of such infections on biomaterial surfaces. However, there has not been an extensive amount of research done on the use of such materials in scaffold fabrication methods based on tissue engineering. Bee pollen has also been found to have anti-inflammatory, anti-bacterial, and anti-viral properties, which can help protect cell-laden biomaterial scaffolds against infection and promote post-operative healing [24]. It can help prevent infection, reduce scarring, and speed up healing post-implantation [159]. The varying quality and quantity of phenolics contribute significantly to a wide range of biological outcomes and antioxidant capacity in bee pollen from various origins. The aforementioned antioxidant potential could prove beneficial for developing tunable platforms that monitor and regulate the presence of oxidative stress after tissue-engineered constructs are implanted.

Stem cells, particularly mesenchymal stem cells (MSCs), form an important research topic in biomedical science due to their healing and regenerative properties [165]. Biomaterials have been used to develop artificial extracellular environments that control the behavior of stem cells [166]. Although the influence of bee pollen in priming cells for augmenting their medicinal effects on stemness, stem cell markers, and proliferation has not been extensively studied, certain studies have indicated that bee pollen may have a positive effect on stemness and stem cell markers. In one such study, bee pollen was found to induce the transcription of stem cell markers, such as Oct4, Nanog, and Sox2, in adipose-derived stem cells (ADSCs) [121]. This was accompanied by an increased expression of proliferation-related genes associated with ADSCs, such as p21 and cyclin D1. Furthermore, it was found that bee pollen supplementation induced the generation of multi-lineage differentiated cells, such as adipocytes, osteoblasts, and chondrocytes. These results suggest that bee pollen may be beneficial in enhancing the stem cell potential of ADSCs as well as increasing their proliferation. In another study conducted by Zhang et al., bee pollen was found to significantly increase the proliferation of human embryonic stem cells (hESCs) [57]. Furthermore, the authors found that the activation of the Wnt/β-catenin signaling pathway mediated this effect. Further research into the underlying mechanisms could allow significant breakthroughs in stem cell research that could have significant implications for medical treatments involving the regeneration of human cells.

## 6. Conclusions

Considering the positive role in preventing infection and cellular damage due to oxidative stress, the well-reported cytocompatibility of bee pollen functionalized materials on fibroblasts, favorable cytoprotective roles on various cell types (e.g., liver, heart), cell-activity-promoting roles on other cell types (e.g., bone), and the positive role on stemness and differentiation of stem cells, future research directed towards bee pollen functionalized materials should be carried out extensively. This would enable researchers to realize and report in-depth knowledge regarding the mechanisms by which bee pollen exerts its bioactivities on cells, thereby increasing awareness in the scientific community for its use in biomaterial fabrication.

This review was intended to be focused on the particular bee product which is bee pollen, a material that was not the subject of focus by Rossi et al. [24], and on defending its suitability in biomaterial fabrication.

In our concluding remarks, we emphasize that including nature-derived bioactive compounds from bee pollen offers a suitable strategy to expand or increase the functional properties of biomaterial scaffolds, broadening their applicability in various biomedical research areas. Hydrogels and electrospun nanofiber scaffolds are currently prevalent in the field of tissue engineering. However, only some studies have looked into the potential of bee pollen as a bioactive functionalization agent, possibly due to the variability mentioned above and a lack of awareness. As one limitation, bee pollen properties vary greatly and are affected by numerous factors, including seasonal collection time and botanical sources. For bee pollen to be successfully utilized for bioengineering purposes, standardizing the dosages of the antimicrobial compounds, typically antioxidants, is necessary. In fact, the extent of control over the biophysical and biological modifications of the scaffolds that are required by various tissues would be positively impacted by the use of standardized preparations or isolated components from bee pollen.

## Figures and Tables

**Table 1:** Antimicrobial and antioxidative properties found in various bee-derived products.

Sl. No.	Bee Products	Bioactive Compounds	Bioactive Properties	References
1	Honey	Flavonoids and phenolic acids like kaempferol, quercetin, chrysin, pinobanksin, luteolin, apigenin, pinocembrin, genistein, hesperetin, *p*-coumaric acid, naringenin, gallic acid, ferulic acid, ellagic acid, syringic acid, vanillic acid, and caffeic acid.	Antimicrobial Anti-inflammatory Antioxidant Anti-aging	[21]
2	Royal Jelly	Flavonoids such as acacetin, apigenin, quercetin, naringenin, isorhamnetin glycosides, kaempferol, formononetin, pinobanksin, luteolin, rutin; phenolic acids such as caffeic acid and ferulic acid; and proteins such as apalbumin, royalactin, royalasin, jellein.	Immunomodulatory Antioxidant Neurotrophic Hypoglycemic Anti-tumor Antimicrobial Anti-inflammatory Anti-allergic Anti-aging Vasorelaxant	[22,23,24]
3	Propolis	Flavonoids such as chrysin, tectochrysin, pinostrobin, apigenin, pinostrobin, chalcone, kaempferol, luteolin, fisetin, quercetin, galangin, pinocembrin, rutin and phenolic acids such as protocatechuic acid, syringic acid, gallic acid, p-coumaric acid, caffeic acid, ferulic acid, artepillin C, chlorogenic acid, and 3,5-dicaffeoylquinic acid.	Antibacterial Antimicrobial Pro-apoptotic Fungicidal Anti-inflammatory Immunomodulatory	[11,12,23]
4	Bee pollen	Flavonoids such as kaempferol, naringenin, quercetin, galangin, pinocembrin, luteolin, apigenin, rutin and isorhamnetin, along with leukotrienes, catechins, and phenolic acids such as chlorogenic acid, protocatechuic acid, syringic acid, gallic acid, p-coumaric acid, caffeic acid, ferulic acid.	Antioxidant Antimicrobial Anti-inflammatory Hypolipidemic Hypoglycemic Anti-diabetic Antiatherogenic Hepatoprotective Cardioprotective	[25]
5	Beeswax	Polyphenols, flavonoids mainly chrysin, and phenolic acids	Antimicrobial Anti-inflammatory Anti-ulcer activity	[26]
6	Bee Bread	Phenolic acids mainly isorhamnetin-*O*-hexosyl-*O*-rutinoside, and flavonoids like Myricetin-3-O-glucoside, quercetin-3-O-rutinoside, kaempferol-3-O-rutinoside, quercetin-3-O-glucoside	Antioxidant Antimicrobial, Anti-tumor, Antihypertensive Neuroprotective	[18,27]
7	Bee Venom	Peptides like melittin, apamin, adolapin, MCD peptide, and enzymes like phospholipase A2 (PLA2), and hyaluronidase.	Antibacterial Anti-inflammatory Anti-cancer, Anti-arthritis Anti-atherosclerotic Antiviral Anti-apoptotic, Anti-diabetic	[28]

**Table 2:** Experimental studies on biomaterials functionalized with bee pollen discussed in this review.

Sr. No.	Building Blocks Polymers	Biomaterial Form	Bee Pollen Form	Major Findings	References
1	Sodium alginate and Polyvinyl alcohol	Electrospun nanofiber	Pristine Bee Pollen	Improved viscosity and conductivity, enabling electrospinning. Incorporation of bee pollen increases the T_g_ value. Possibility for the presence of antimicrobial, antioxidant, and anti-inflammatory properties for use in wound dressings.	[102]
2	Chitosan	Freeze Dried Film	Pristine Bee Pollen	Increased polyphenol content in films. High antioxidative potential Antibacterial properties against *Listeria monocytogenes*, *Salmonella sp.*, *Escherichia coli*, and *Staphylococcus aureus.*	[103]
3	Gelatin, and Beetosan^®^	Hydrogel	Pristine Bee Pollen	Improved homogeneity on the biomaterial surface. Improved wettability on a biomaterial surface. Cytocompatible effect on WEHI 164 (fibroblast) cell lines anti-cancerous effect on Jurkat (cancerous) cell lines	[104]
4	Gelatin, sodium carboxymethyl cellulose	Hydrogel	Bee Pollen Powder	Biocompatible material shows no signs of skin irritation in rabbits. Superior wound healing activity with 95% wound closure after 25 days	[105]
5	Casein, and Starch	Film	Pristine Bee Pollen	Incorporation of bee pollen improves thermal and mechanical properties.	[106]
6	Polyacrylic acid	Hydrogel	Pristine Bee Pollen	High water uptake ability against Ringer’s solution, simulated body fluid (SBF), and pseudo-extracellular fluid (PECF). Improved bio-stability in physiological and wound environment conditions.	[107]
7	Hollow Pollen Grains (HPGs)	Scaffold	Nature-made Pollen Grains (PGs) of *Pistacia* *vera L*.	Suitable for encapsulating bone morphogenetic protein 4 (BMP4). Provided cell adhesion sites for hAD-MSCs and maintained their viability. High osteogenic capability, as evidenced by increased ALP activity and expression of RUXN2 and OSC.	[108]
8	Alginate and Hyaluronic acid	Hydrogel Bioink	Pristine Sunflower Pollen	Improved printability and reduced nozzle clogging. Improved mechanical stability with self-standing scaffolds. Cytocompatibility with 94% viability of Huh-7 cells.	[109]
9	Bovine Serum Albumin	Nanoparticle	Ethanol extract of *Trifolium alexandrinum* Bee Pollen	Enhancement of anti-cancer properties in a dose-dependent manner. Improved anti-proliferative outcome with combinational therapy using Avastin to downregulate HRAS and MAPK pathways with simultaneous upregulation of apoptotic genes	[110]
10	Bovine Serum Albumin, and Protamine	Hydrogel nanoparticle	Pristine form (Solution)	Improved the anti-cancer effectiveness of bevacizumab against A549 and MCF-7 cell lines, in addition to reducing the dosage of the drug.	[111]

## Data Availability

The data presented in this study are available on request from the corresponding author.
